# Rescuing the Right Ventricle: Mechanical Support After Pediatric Heart Transplantation

**DOI:** 10.1016/j.atssr.2023.12.004

**Published:** 2023-12-27

**Authors:** Amee M. Bigelow, Catherine Kapcar, Eric Lloyd, Lydia K. Wright, Benjamin A. Blais, Jordan Voss, Ashley B. Walczak, Matthew Deitemeyer, Vicky Duffy, Deipanjan Nandi, Patrick I. McConnell

**Affiliations:** 1 Department of Pediatrics, The Heart Center, Nationwide Children’s Hospital, The Ohio State University, Columbus, Ohio; 2 Department of Cardiothoracic Surgery and Perfusion Services, The Heart Center, Nationwide Children’s Hospital, Columbus, Ohio

## Abstract

**Background:**

Right ventricular (RV) failure after heart transplantation (HT) is common in those with pretransplantation elevated pulmonary vascular resistance (PVR). Mechanical circulatory support has been used as a bridge to recovery, with mixed outcomes. We describe a patient with failed single-ventricle palliation in whom severe RV failure developed after HT. We review the current literature and outline our post-HT strategy.

**Methods:**

An infant with trisomy 21, severely unbalanced right dominant atrioventricular septal defect, and hypoplastic aortic arch was palliated with a hybrid procedure. At 6 months of age, cardiac catheterization measured PVR index of 5.47 Wood units × m^2^ on maximal medical therapy. The patient was deemed unsuitable for second-stage palliation and underwent HT at 18 months of age. Despite preemptive medical therapies, acute RV failure developed, necessitating extracorporeal membrane oxygenation. He was quickly converted to main pulmonary artery to left atrial cannulation. Unloaded RV function normalized; he was weaned from support and discharged home 5 weeks after HT.

**Results:**

Failure of medical therapy in RV failure after HT requires escalation to mechanical circulatory support. We review the literature on RV failure and support after HT. We also describe a novel cannulation strategy to provide a reliable way to directly reduce RV afterload, to allow physiologic training of the right ventricle to a higher PVR, and to maintain normal left ventricular coupling and loading.

**Conclusions:**

In pediatric patients with elevated PVR undergoing HT, advanced therapies can be used effectively to treat acute RV failure. Unique cannulation strategies may play a role in improving survival of similar patients.

## Visual Abstract

**Figure:**
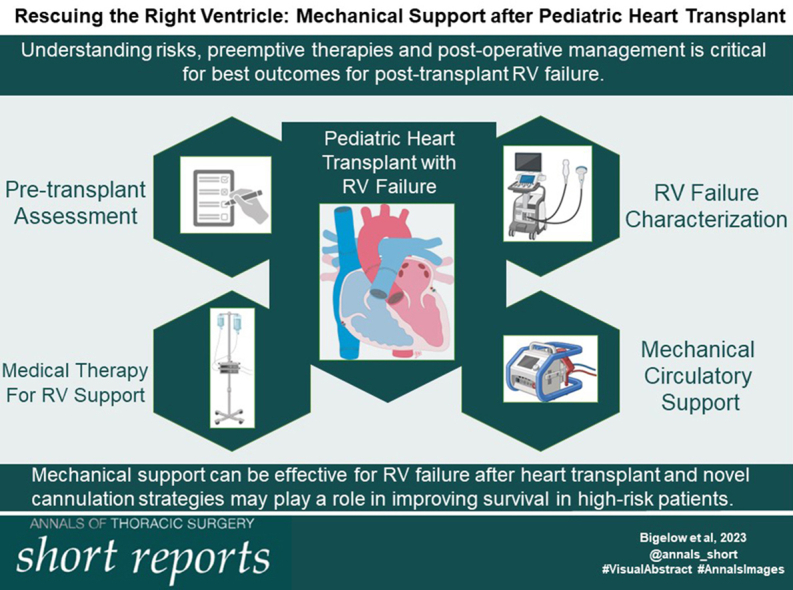

**In Short**

▪ Mechanical circulatory support, often a final recourse, is a crucial intervention for pediatric patients experiencing acute right ventricular (RV) failure after heart transplantation.
▪ Novel cannulation strategies that directly reduce RV afterload, facilitate RV adaptation to higher pulmonary vascular resistance, and maintain normal left ventricular loading conditions can be effective approaches.
▪ Innovative cannulation techniques have potential for improving survival in high-risk patients with elevated pulmonary vascular resistance undergoing heart transplantation.

One-year survival after pediatric heart transplantation (HT) is now as high as 85% to 90%,^1^ allowing consideration of higher risk patients like infants with chromosomal abnormalities and varying degrees of pulmonary hypertension. Acute right ventricular (RV) failure remains a significant cause of morbidity and mortality after HT.^2^ In adult series, RV failure accounts for up to 50% of all cardiac complications and 19% of early posttransplantation deaths.^3^

The incidence of RV failure in pediatric HT is unknown, but elevated pulmonary vascular resistance (PVR) before HT significantly increases post-HT mortality.^3^ Risk factors for RV failure in pediatric patients, especially those with congenital heart disease, include elevated PVR, reperfusion injury, graft adaptation to the recipient hemodynamic requirements, and prolonged ischemia time, among others.^4^

Effective medical strategies target RV support, but in severe, refractory RV failure, mechanical circulatory support (MCS) serves as a bridge to recovery. One-year survival rates for pediatric posttransplantation extracorporeal membrane oxygenation (ECMO) patients range from 62% to 67%, with limited data on ECMO in acute RV failure.^5^ We herein discuss RV failure and its causes and medical treatments, MCS after HT, and alternative cannulation approaches. Specifically, we present the case of a patient with failed single-ventricle palliation in whom post-HT RV failure developed as a result of elevated PVR index (PVRi). We detail a unique post-HT RV MCS approach and explore modalities to improve high-risk RV failure outcomes after HT.

## Patients and Methods

A male infant born with trisomy 21, a severely unbalanced right dominant atrioventricular canal defect, hypoplastic aortic arch, and parachute left atrioventricular valve underwent a hybrid palliation procedure with a patent ductus arteriosus stent and bilateral branch pulmonary artery (PA) banding (3.5 mm) in the neonatal period. At 6 months of age, despite medical optimization with pulmonary vasodilator therapies (sildenafil and macitentan), diagnostic catheterization revealed elevated PA pressures and transpulmonary gradient and a high PVRi at 5.47 Wood units × m^2^. He was not thought to be a suitable candidate for a second-stage palliation. Subsequent attempts to mitigate the PVR were unsuccessful, leading to his listing for HT.

After 328 days on the waitlist, a suitable heart became available. The patient underwent an orthotopic HT with bicaval anastomosis, aortic arch reconstruction, and bilateral branch PA arterioplasty and reconstruction (272 minutes of cardiopulmonary bypass time, 263 minutes of organ cross-clamp time, and 47 minutes of antegrade cerebral perfusion). The intraoperative course was uneventful, with final transesophageal echocardiography demonstrating normal RV function with greater than half-systemic RV pressure estimates.

## Results

Despite preemptive medical therapies in the early postoperative hours, inadequate indices of perfusion developed. Echocardiography revealed severe tricuspid regurgitation, severe qualitative RV systolic dysfunction, close to systemic RV pressures, and preserved left ventricular (LV) systolic function. Lung compliance was adequate. The decision was made to centrally cannulate to venoarterial ECMO support using a 12F DLP single-stage venous cannula with right angle metal tip (Medtronic) for the right atrium and a 10F Bio-Medicus one-piece (Medtronic, Inc., Minneapolis, MN) for the aortic cannula with a PediMag (Abbott Inc, Pleasanton, CA) blood pump and QUADROX-iD pediatric oxygenator (Getinge AB, Goteborg, Sweden). On postoperative day 2, mild bilateral branch PA stenoses were noted, worse on the left side, requiring a 16-mm Mega LD stent (Medtronic).

**Figure 1:**
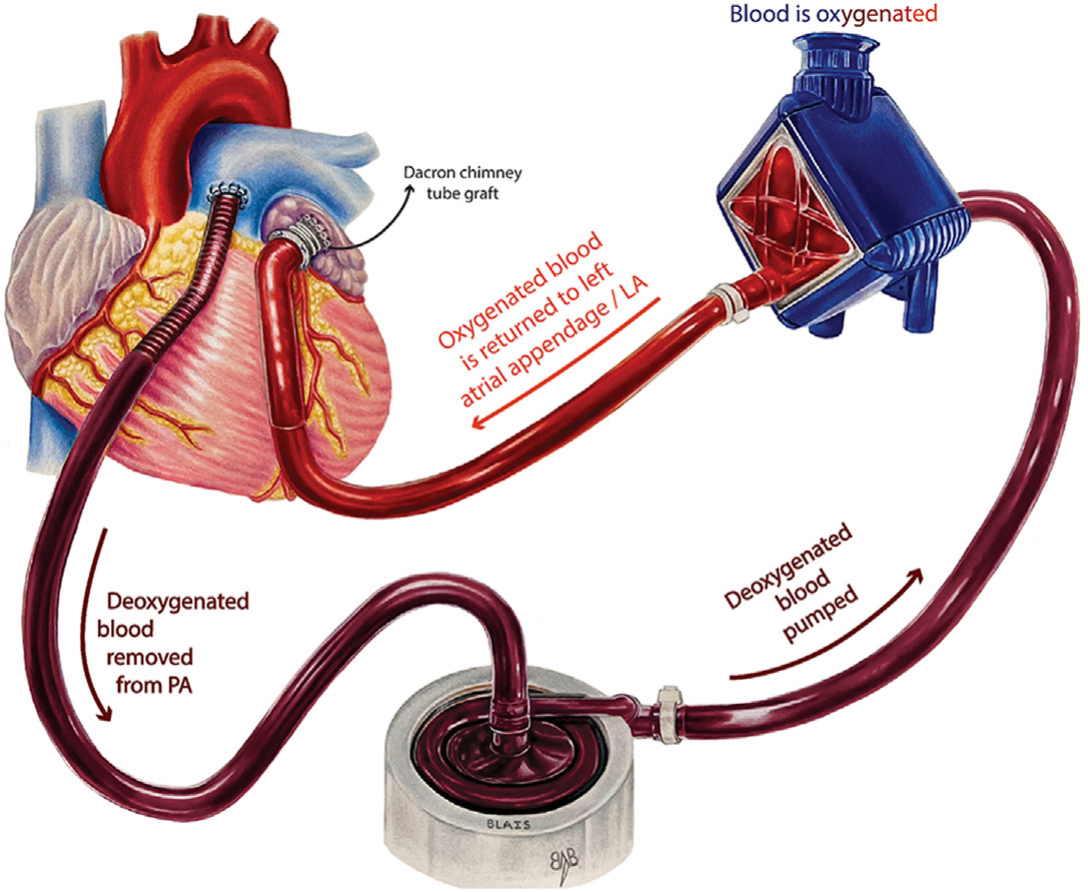
Schematic for pulmonary artery (PA) to left atrium (LA) extracorporeal membrane oxygenation. Note: In addition to the depicted setup, a Dacron chimney tube graft can be employed for improved cannula positioning.

**Figure 2:**
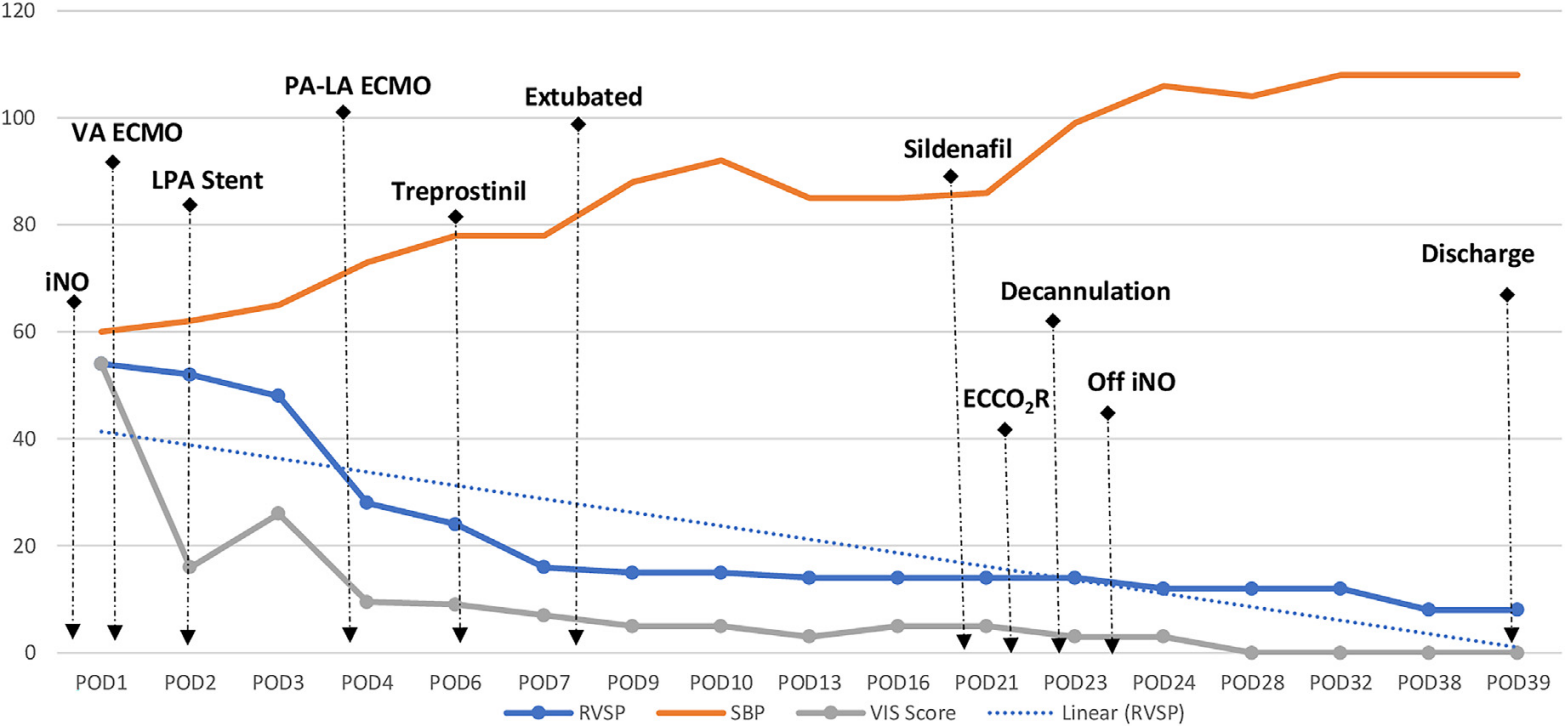
Echocardiographically obtained right ventricular systolic pressure (RVSP) measurements using the tricuspid regurgitation jet are shown (solid blue line). Corresponding simultaneous systolic blood pressure (SBP) measurements are shown by the orange line. In addition, the vasoactive inotropic score (VIS) is shown on the gray line. Linear trend line for the RVSP measurement is shown by the dashed blue line. Significant events are shown by dashed black arrows. (ECCO_2_R, extracorporeal carbon dioxide removal; ECMO, extracorporeal membrane oxygenation; iNO, inhaled nitric oxide; LPA, left pulmonary artery; PA-LA, pulmonary artery–left atrium; POD, postoperative day; VA, venoarterial.)

Although end-organ function was improved and structural issues were addressed, the right ventricle remained hypertensive and dysfunctional. To address the persistent RV hypertension and dysfunction, on postoperative day 4, we opted to directly reduce RV afterload to facilitate RV training to function with his PVR through PA to left atrium (LA) cannulation (Figure 1). In the operating room, we tunneled a 16Fr DLP one piece wire-wound arterial cannula along the right costal margin with placement into the left atrium, then a 16Fr DLP metal-tip right angle cannula was inserted into the main PA. After cannula placement, a blood-primed circuit was connected to the PA as the inflow (venous) and to the LA as the return (arterial) with a Quadrox iD pediatric oxygenator and a PediMag blood pump. This setup allowed immediate improvement in RV systolic function, as confirmed by postoperative transesophageal echocardiography. Serial RV systolic pressure measurements and significant events are summarized in Figure 2.

Intensive care unit length of stay was 30 days and hospital length of stay was 169 days, including preoperative admission, and he was discharged on postoperative day 38 with normal biventricular function, trivial tricuspid regurgitation, and no evidence of RV hypertension. He was prescribed sildenafil and subcutaneous treprostinil, standard immunosuppression, diuretics, and infectious prophylactics. By 1 year after HT, treprostinil and sildenafil were successfully discontinued, maintaining stable cardiac filling pressures and a normal PVRi of 2 Wood units × m^2^. The patient remains asymptomatic and active and is achieving developmental milestones. Characteristics of the patient are summarized in the Supplemental Table.

## Comment

We report a successful case of PA-to-LA ECMO support in a pediatric patient with RV failure despite multiple risk factors, such as elevated pretransplantation PVR, prolonged ischemia time (>4 hours), and congenital heart disease. RV failure after HT is a well-recognized concern in both adult and pediatric patients, resulting in substantial morbidity and mortality.^3^^,^^6^

RV failure after transplantation can be attributed to various anatomic and physiologic factors. Our understanding of the molecular mechanisms underlying RV dysfunction remains limited. Unlike the left ventricle, the right ventricle exhibits unique structural, loading conditions and cellular-level distinctions.^7^ The right ventricle’s adaptive response to pressure- or volume-loaded conditions along with ischemia associated with congenital heart disease is unique.^7^^,^^8^ As a low-pressure, high-volume chamber, the right ventricle is highly sensitive to changing loading conditions; its thin walls allow accommodation to significant volume increases without altering diastolic wall stretch, filling at or below its unstressed volume. RV afterload relies on pulmonary arterial elastance, which governs RV-arterial coupling. Increased RV afterload, common in PA hypertension, challenges its adaptive mechanisms, relying more on RV contractility, and can have an impact on ventriculoventricular interactions, leading to leftward septal shift, reduced LV filling, and compromised cardiac output.

Understanding the intricacies and potential for failure, we now pivot to the critical realm of pretransplantation assessment.

### Pretransplantation Assessment

Preoperative assessment involves cardiac catheterization to evaluate cardiac filling pressures, PA pressures, pulmonary capillary wedge pressures and calculation of PVRi. Elevated PVR is common in restrictive cardiomyopathy and failing single-ventricle physiology.^9^ In congenital heart disease, often considered is the use of cardiac magnetic resonance imaging for precise assessment of differential right and left PA flow. However, a calculated PVR may not be accurate in all physiologies. Reliable predictors of RV failure in children, particularly in situations of low cardiac output, are not well defined.^9^ Some adult studies suggest PVRi >6 Wood units × m^2^, systolic PA pressure ≥60 mm Hg, or a transpulmonary gradient ≥15 mm Hg as high-risk factors for RV failure postoperatively. One pediatric study suggested a PVRi of ≥9 Wood units × m^2^ as a practical threshold for identifying high-risk pediatric patients.^10^ Whereas individual parameters alone lack prognostic value, they aid in risk assessment and guide patient management. Informed decision-making requires precise interpretation of diagnostic studies and multidisciplinary collaboration.

### Defining RV Failure

Defining RV failure in pediatrics is challenging because of unreliable imaging parameters, unvalidated for pediatrics, and often lack of consistent direct pressure monitoring. No consensus definition exists, so a combination of hemodynamic assessment, clinical judgment, examination, and echocardiographic findings is used.

Impending RV failure can be identified by a central venous pressure exceeding 15 mm Hg or unresponsiveness to volume challenges. Interpretation of central venous pressure can be complicated by factors like severe tricuspid regurgitation, RV failure, superior vena caval obstruction, and high ventilatory pressures. In high-risk patients, use of dedicated right-sided pressure monitoring lines should be considered. Examination findings and laboratory results support clinical diagnosis. Echocardiographic interpretation after transplantation is challenging. Qualitative RV systolic function and LV filling are commonly used as surrogates for RV dysfunction. Less frequently used are objective echocardiographic markers like tricuspid annular plane systolic excursion, RV performance index, and peak longitudinal strain for measuring RV function.

### Medical Therapy for RV Support

In high-risk cases, perioperative risk mitigation involves optimizing preoperative end-organ function, careful myocardial protection during procurement, and minimizing ischemia time. Postoperatively, the focus shifts to distinct medical management for RV failure, prioritizing preload, reducing PVR, and avoiding inappropriate inotropic support or significant LV afterload reduction. Optimizing myocardial oxygen delivery, limiting ventricular oxygen consumption, and reducing RV afterload by optimal ventilatory management are crucial. Consideration is given to proactive pulmonary vasodilators, even in the operating room, to achieve PVR reduction without a significant drop in systemic vascular resistance. Whereas limited evidence guides vasoactive and inotropic use, goals should maintain systemic blood pressure for adequate coronary perfusion, enhance RV contractility, and optimize RV diastolic filling by controlling heart rate. Addressing potential causes of RV failure, including infection, postcardiotomy inflammatory response, arrhythmias, and acute rejection, is essential.

### Mechanical Circulatory Support for the Right Ventricle

Timely initiation of MCS to prevent end-organ damage and to facilitate recovery is crucial. In cases of extreme risk or early signs of RV failure, a decision for MCS in the operating room may be warranted.

MCS options for RV failure depend on the patient’s needs. Venoarterial ECMO addresses short-term post-HT complications such as postcardiotomy inflammation, arrhythmias, rejection, and infection. Although it enhances RV unloading, it does not aid RV recovery or facilitate assessment. Venovenous ECMO suits hypoxemia but lacks RV support. Newer lung assist devices use a low transmembrane pressure oxygenator employing an arteriovenous configuration for carbon dioxide removal and oxygenation, potentially unfit for untrained or dysfunctional RVs. PA-to-LA ECMO offers the most physiologic support. The PA cannula directly offloads the right ventricle, enabling it to flow to both the pulmonary bed and the pump, permitting gradual workload transfer to a higher PVR, whereas the LA cannulation maintains LV coupling and loading. Initially, the RV may preferentially use the oxygenator because of lower resistance. However, as the pulmonary bed is treated, more blood flows through it, enabling direct RV function assessment. This strategy offers central cannulation for mobility and avoids a ventriculotomy. The patient’s survival was significantly improved by employing a strategic cannulation approach, complemented by the judicious use of pulmonary vasodilator therapies and posttransplantation stabilization. Importantly, the targeted use of pulmonary vasodilators played a pivotal role in alleviating the strain on the right ventricle. Recognizing that pulmonary hypertension would not resolve immediately, the cannulation strategy provided a foundation for sustained support. This allowed the RV to gradually adapt to elevated pressures while titrating pulmonary vasodilator therapies and allowing PAs time for positive remodeling. Whereas this comprehensive approach emphasizes the multifaceted nature of ensuring the patient’s successful recovery, it also underscores the ongoing challenges and areas that warrant further investigation. The need for a larger sample size becomes apparent to assess unique MCS configurations in this group of patients compared with conventional methods.

### Future Directions

Understanding the physiologic and molecular mechanisms of RV dysfunction and remodeling is still in its early stages. Establishing specific mechanisms for the vulnerability of the right ventricle in patients with congenital heart disease is crucial for developing novel therapies that could address and mitigate the acute mechanisms contributing to RV failure.^7^

### Conclusion

Preoperative identification of high-risk patients undergoing HT can optimize management of postoperative RV failure. Pediatric patients with elevated PVR can successfully undergo HT and be supported postoperatively to survival. MCS can be used effectively to treat patients with RV failure after transplantation, and novel cannulation strategies may play a role in improving survival in high-risk patients.
