# Supplementary material for: Rescuing the Right Ventricle: Mechanical Support After Pediatric Heart Transplantation
Source: Ann Thorac Surg Short Rep. 2023 Dec 27;2(2):277–81. doi: 10.1016/j.atssr.2023.12.004 (PMC11708400; doi:10.1016/j.atssr.2023.12.004)
Supplement: Supplementary Table [file mmc1.docx]

**Supplemental Table 1: Patient Characteristics:**

| **Characteristic** |  |
| --- | --- |
| **Age at transplant** | **18 months** |
| **Weight at transplant** | **11.5 kg** |
| **Donor weight** | **9.7 kg** |
| **Pre-ECMO creatinine** | **0.28 mg/dL** |
| **Peak creatinine, mg/dL** | **2.05 mg/dL (POD3)** |
| **Pre-ECMO Total bilirubin** | **0.4 mg/dL** |
| **Peak AST** | **5294 U/L (POD4)** |
| **Peak ALT** | **1910 U/L (POD3)** |
| **PRA at transplant, %** | **10** |
| **Pre-transplant PVRi** | **5.47 WUxm^2^** |
| **Ischemic time, min** | **263** |

**ECMO: Extracorporeal Membrane Oxygenation, PRA: Panel-reactive antibody, AST: aspartate aminotransferase , ALT: alanine transaminase, PVRi: Pulmonary vascular resistance index**
